# The impact of platelets on the metastatic potential of tumour cells

**DOI:** 10.1016/j.heliyon.2024.e34361

**Published:** 2024-07-14

**Authors:** Hans Raskov, Adile Orhan, Mette Ørskov Agerbæk, Ismail Gögenur

**Affiliations:** a Center for Surgical Science, Zealand University Hospital, Køge, Denmark; b University of Copenhagen, Faculty of Health and Medical Sciences, Copenhagen, Denmark; c Centre for Translational Medicine and Parasitology, Department of Immunology and Microbiology, University of Copenhagen, Denmark; d Department of Clinical Medicine, University of Copenhagen, Copenhagen, Denmark

## Abstract

In cancer, activation of platelets by tumor cells is critical to disease progression. Development of precise antiplatelet targeting may improve outcomes from anticancer therapy.

Alongside a distinct shift in functionality such as pro-metastatic and pro-coagulant properties, platelet production is often accelerated significantly early in carcinogenesis and the cancer-associated thrombocytosis increases the risk of metastasis formation and thromboembolic events. Tumor-activated platelets facilitate the proliferation of migrating tumor cells and shield them from immune surveillance and physical stress during circulation. Additionally, platelet-tumor cell interactions promote tumor cell intravasation, intravascular arrest, and extravasation through a repertoire of adhesion molecules, growth factors and angiogenic factors. Particularly, the presence of circulating tumor cell (CTC) clusters in association with platelets is a negative prognostic indicator.

The contribution of platelets to the metastatic process is an area of intense investigation and this review provides an overview of the advances in understanding platelet-tumor cell interactions and their contribution to disease progression. Also, we review the potential of targeting platelets to interfere with the metastatic process.

## 1 Background

Metastasis, the predominant contributor to cancer-related deaths, encompasses a complex sequence of regulated events including migration, intravasation, circulation, intravascular arrest, and extravasation of tumor cells, excellently reviewed by Gerstberger et al. [1]. An essential phase of the metastatic cascade involves the presence of circulating tumor cells (CTCs) navigating the bloodstream. This is a vulnerable process where most CTCs are rapidly eliminated by cytotoxic immune cells and the fluidic shear forces encountered within the vascular system. The multifaceted roles played by platelets in promoting oncogenesis are underscored by the protection of CTCs against physical stress and cytotoxic immune reactions within the circulation [2,3].

Platelet surfaces are densely coated with receptors and adhesive proteins that enable them to interact with other cells and respond to a wide variety of external cues [4]. The key role for commonly activated platelets lies in hemostasis where they prevent bleeding by binding to damaged blood vessels and stabilize thrombus formation by extrinsic and intrinsic coagulation pathways. Activation is normally induced by factors released from damaged endothelium. In cancer, however, the direct uptake and storage of tumor-secreted factors has a significant impact on platelet activation, production, and function [4]. Circulating tumor-activated platelets (TAPs) are associated with an increased risk of metastasis and poor prognosis in various types of cancer [5,6]. Also, TAPs, are considered to be responsible for the hypercoagulable state and thromboembolic events observed in cancer patients [7,8].

**Fig. 1:**
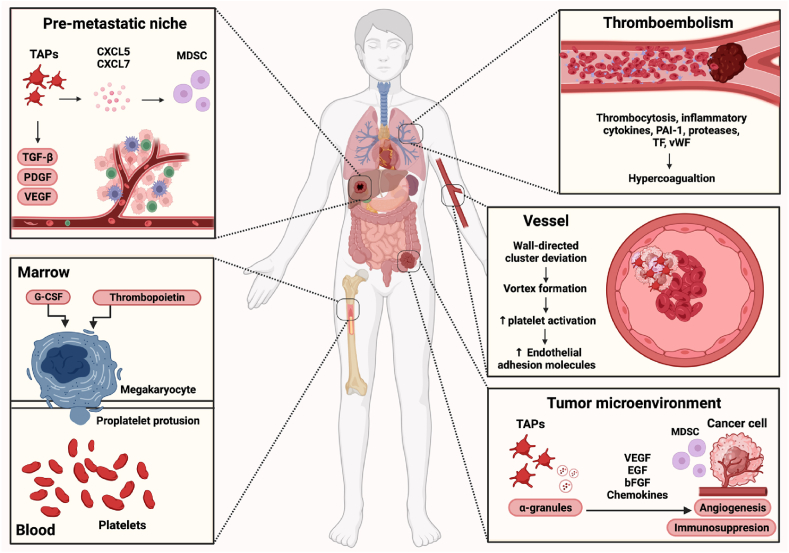
Platelets are key mediators in both the early and late stages of cancer. Top-down from left: In forming the PMN, TAPs produce chemokines such as CXCL5 and CXCL7 to recruit protumorigenic immune cells including neutrophils and MDSCs to the PMN. In addition, TAPs secrete growth factors such as VEGF, PDGF and TGF-β to facilitate the formation and progression of PMN. In the bone marrow, thrombopoietin and G-CSF can boost platelet production more than 20 times, leading to thrombocytosis. Cancer-associated thrombocytosis and increased levels of inflammatory cytokines, proteases, PAI-1, TF, and vWF lead to hypercoagulation and thromboembolism. In the bloodstream, activated platelets may form clusters with cancer cells and shield them from immune-mediated elimination and physical stress. Due to the wall-directed deviation, CTCs clusters approach the vessel wall, creating a vortex of plasma and shear force that activate platelets and endothelial adhesion molecules, easing the arrest and intravasation process (Fig. 2). In the TME, TAPs release α-granules containing chemokines, growth factors and clotting factors, promoting angiogenesis. Platelet-derived chemokines attract MDSCs to promote immune suppression. bFGF: basic fibroblast growth factor. CTCs: Circulating tumor cell. CXCL5: Chemokine (C-X-C motif) ligand 5. CXCL7: Chemokine (C-X-C motif) ligand 7. G-CSF: Granulocyte colony-stimulating factor. MDSC: Myeloid-derived suppressor cell. PAI-1: plasminogen activator inhibitor-1. PDGF: Platelet-derived growth factor. PMN: premetastatic niche. TAPs: Tumor-activated platelet. TF: tissue factor. TGF-β: Transforming growth factor-beta. TME: Tumor microenvironment. VEGF: Vascular endothelial growth factor. vWF: von Willebrand Factor. Figure made on biorender.com.

This review aims to provide an overview of our current knowledge of platelet activation and its role in disease progression (Fig. 1). Advances in understanding the platelet/tumor cell interactions and their contribution to the metastatic process may accelerate the development of improved therapeutic strategies.

**Fig. 2:**
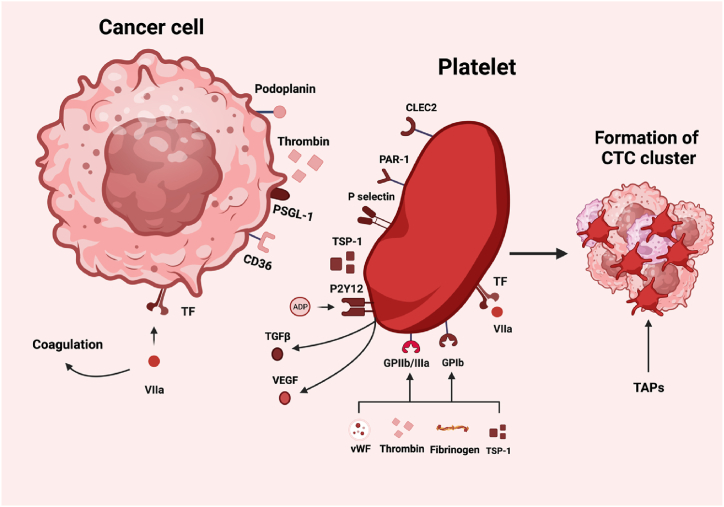
Major receptors and ligands on platelets and tumor cells involved in hyper-coagulation and the formation of CTCs clusters and tumor cell/platelet/leukocyte aggregates. VIIa: coagulation factor VIIa. ADP: adenosine diphosphate. CLEC-2; C-type lectin-like receptor 2. CTCs: circulating tumor cells. P2Y12: purinergic receptor P2Y, G-protein coupled, 12 protein. PAR-1: Protease-activated receptor-1. PSGL-1: P-selectin glycoprotein ligand-1. TF: tissue factor. TGFβ: Transforming growth factor β. TSP-1: Thrombospondin 1. vWF: von Willebrand Factor. Figure made on biorender.com.

### 1.1 Cancer-associated thrombocytosis and thromboembolism

In the bone marrow of healthy individuals, megakaryocytes extend proplatelet protrusions into sinusoidal spaces and release vast numbers of platelets into circulation [9] (Fig. 1) at a rate of approximately 100 billion platelets per day. Circulating for 8–9 days, the physiological range is 150 to 400x10^9^ platelets/L of blood. Exceeding this level is defined as thrombocytosis. In response to tumor-secreted factors such as IL-6-stimulated thrombopoietin, granulocyte colony-stimulating factor, and granulocyte-macrophage colony-stimulating factor, platelet production can increase more than 20-fold [10] and platelets from patients with metastatic cancers show significantly increased aggregation in response to tumor-derived agonists [11].

Cancer-associated thrombocytosis often predicts poor outcomes as the motility and invasiveness of cancer cells are enhanced through platelet crosstalk with cytotoxic immune cells such as NK and T cells [12,13].

Activated platelets increase the expression of the TGF-β-docking receptor GARP (Glycoprotein A Repetitions Predominant) and release granules containing a variety of signaling molecules including TGF-β, platelet factor 4, serotonin and proteases. A preclinical study demonstrated that platelet-derived GARP-TGF-β complexes were the primary cause of platelet-derived T-cell suppression and promotion of tumour growth [14].

However, platelet counts alone may not be an independent prognostic factor for overall survival. The predictive value is improved when combined with additional factors such as the platelet/lymphocyte ratio [15], hemoglobin, albumin, and total leukocyte counts [16]. Patients with elevated platelet/lymphocyte ratios have an increased risk of distant metastases and worse prognoses as shown for head and neck squamous cell carcinoma [17], nasopharyngeal carcinoma [18], oral cancer [19], gastric cancer [20], renal cell cancer [21], colorectal cancer (CRC) [22], bladder cancer [23], and cervical cancer [24].

Cancer-associated thrombocytosis heightens the risk of thromboembolism (TE). Approximately 15–30 % of patients with cancer, especially of the central nervous system, pancreas, lung, stomach, and hematological cancers, suffer from TE [25] and the risk is further exacerbated by surgery, chemotherapy, and therapies involving anti-angiogenic and hematopoietic growth factors [26].

Additionally, an increased risk of TE during immune checkpoint inhibitor treatment has been reported and as many as 10 % may experience TE during the first 12 months of treatment [27]. Upon lifting the suppression on cytotoxic T cells, the release of cytokines triggers inflammation, activation of endothelial cells and neutrophil discharge of extracellular traps (NETs), all contributing to thrombosis [28].

Pre-treatment platelet counts, the platelet/leukocyte ratio, and body mass index can help assess a patient's risk of TE. However, the risk of TE in cancer patients is not determined by the level of thrombocytosis but indeed by the functional alterations of TAPs [29]. As an example, a recent study demonstrated platelet uptake of tumor-derived extracellular vesicles resulting in efficient activation and accelerated thrombosis formation [30].

Furthermore, other cancer-related molecular compounds such as neutrophil extracellular traps, circulating inflammatory cytokines, proteases (including cancer procoagulant), plasminogen activator inhibitor-1 (PAI-1), and tissue factor (TF) together with endothelial dysfunction significantly contribute to the heightened risk of cancer-associated TE and disseminated intravascular coagulation [[31], [32], [33]].

In mouse models, human blood samples, and melanoma tissue samples, the tumor cell-mediated release of von Willebrand Factor (vWF) from platelets and endothelial cells resulted in platelet aggregation and accelerated deposition of thrombin within the melanoma vasculature [34]. Here, it was suggested that inhibition of the proteolytic activity of the vWF-cleaving protease ADAMTS-13.

(a disintegrin and metalloproteinase with a thrombospondin type 1 motif, member 13) accounted for the hyper-coagulatory state [34]. Some patients with advanced cancer are deficient in ADAMTS-13 resulting in an increased risk of TE and disease progression [35].

Some cancers, especially hematologic cancers, may release cytokines like IL-1, IL-6, and TNF-α, that negatively impact megakaryocytes and suppress platelet production, explaining some cases of cancer-associated thrombocytopenia. In a retrospective cohort study, analysis of severe thrombocytopenia (platelet count <50,000/μL) revealed a prevalence of 7 % (95 % CI 6%–8%) in patients diagnosed with solid tumors and 30 % (95 % CI 27%–34 %) in patients with hematologic malignancies. Other causes of significant thrombocytopenia included tumor involvement of bone marrow and spleen, venous TE, disseminated intravascular coagulation, thrombotic thrombocytopenic purpura or hemolytic uremia syndrome [36].

In advanced solid tumors, thrombocytopenia can arise from several factors, including liver dysfunction, spleen enlargement, and chemotherapy-induced bone marrow suppression. Additionally, inflammation within the TME and/or TE can contribute to increased platelet consumption through inflammatory mediator release and enhanced platelet aggregation.

### 1.2 Platelet activation by tumor cells

Platelets are activated by direct or indirect interaction with tumor cells (Fig. 2), resulting in the release of granule-stored substances, increased aggregation, and an altered RNA profile [27].

Specific changes relating to tumor activation that may serve as potential biomarkers for staging, and prognostication include activated integrin α2b-β3, lysosomal-activated membrane protein (CD63), P-selectin (CD62P), and CD24 that may be detected by flow cytometry together with platelet-specific antigens such as the glycoprotein complexes Ib-IX-V, GPIIb, and GPIIIa [37].

Activated platelets contain different types of RNAs, including precursor- and mature messenger RNA (mRNA), transfer RNA, microRNA, and other non-coding RNAs. Studies employing amplification and sequencing of platelet-derived RNA in patients with various localized and metastatic cancers (e.g., NSCLC, colorectal, glioblastoma, pancreatic, hepatobiliary, and breast) have identified distinct RNA profiles. These profiles demonstrate high accuracy in both differentiating cancer patients from healthy controls (95 % accuracy) and predicting the specific tumor type (97 % accuracy) [38].

Two independent studies, one focusing on glioblastoma [39] and another on breast cancer [40], have provided compelling evidence for the existence of unique RNA signatures that distinguish cancer patients from healthy controls. However, further validation using larger patient cohorts remains necessary to evaluate the generalizability and reliability of these signatures [39,40].

The platelet activation primarily relies on phosphorylation of the immunoreceptor tyrosine-based activation motifs (ITAMs) of the platelet receptors shown in Fig. 2.

Tumors can secrete adenosine diphosphate (ADP) that activates the platelets P2Y12 receptor to secure stable adhesions with tumor cells as reviewed in Ref. [41] and leads to degranulation and release of bioactive molecules, including the vascular endothelial growth factor (VEGF) released from α-granules. Furthermore, ADP activates GPIIb/IIIa, a platelet surface integrin complex and receptor for von Willebrand factor and fibrinogen, that triggers platelet aggregation.

Thrombin, a protease synthesized in the liver as a zymogen glycoprotein, may also be produced by tumor cells and plays a pivotal role in platelet activation. In concert with tissue factor/coagulation factor 3, also known as thromboplastin, which is highly expressed by many tumors [[29], [30], [31]], thrombin drives subsequent events of the coagulation process via the platelet protease-activated receptor-1 (PAR-1) that trigger intracellular signals leading to platelet activation. Further, thrombin contributes to the formation and reorganization of the extracellular matrix (ECM) and may cause dysregulation of the endothelial barrier through the loss of intercellular adherens junctions [42]. Yet another abundant platelet adhesion mechanoreceptor, the GPIb-IX-V glycoprotein complex, serves as a binding site for various ligands, including thrombin and vWF. This complex not only participates in platelet adhesion but also plays a role in thrombopoietin secretion and platelet production [43]. The pro-coagulatory vWF is released from platelet α-granules and from tumor cells. vWF is considered a promotor of inflammation and an important mediator of tumor cell/platelet aggregation and metastasis [44] among others by increasing vascular permeability and facilitating adherence and endothelial transmigration of CTCs [45].

### 1.3 Platelets are storage sites for pro-angiogenic factors

Comprising approximately 10 per cent of the platelet volume, 50–80 α-granules in each platelet harbor a mixture of more than 300 distinct cytokines, chemokines, growth factors, and adhesive and clotting factors [46]. Importantly, α-granules serve as a primary reservoir for angiogenic factors such as VEGF, platelet-derived growth factor (PDGF), epidermal growth factor (EGF), and basic fibroblast growth factor (bFGF) [47,48] through which, platelets stimulate angiogenesis in the tumor microenvironment (TME). Platelets can directly take up tumor cell-secreted VEGF and increase their VEGF storage [49] and upon activation by tumor-secreted factors, such as ADP and tissue factor, platelets release the α-granule-stored angiogenic factors to stimulate angiogenesis and tumor growth. Pulling in the opposite direction, and when stimulated by thromboxane A_2_, α-granules can downregulate angiogenesis within the tumor microenvironment (TME) by releasing a repertoire of anti-angiogenic factors, including angiopoietin-1 (ANGPT1), sphingosine 1-phosphate (S1P), thrombospondin-1 (TSP1), and endostatin [6].

### 1.4 Platelets support tumor cell invasion and migration

In response to hypoxia and tumor-released signalling molecules, platelets extravasate and infiltrate the tumor microenvironment. The interplay between platelets and tumor cells suppresses antitumor immune responses and orchestrates a priming mechanism fostering metastasis formation. Platelets, being an important contributor of transforming growth factor-beta (TGF-β), initiate the activation of TGF-β/Smad and NF-κB signaling pathways in tumor cells resulting in their transition to a motile, invasive mesenchymal-like phenotype, a process called endothelial-mesenchymal transition or EMT. The EMT promotes tumor cell migration towards vessels by following growth factor and cytokine gradients and aided by motile tumor-associated macrophages. Crossing the endothelial barrier, these tumor cells intravasate with an enhanced potential to metastasize [50].

Platelets release membrane-derived microparticles, being particles 100 nm to 1 μm in diameter and covered by a lipid bilayer mirroring the platelet exterior. The internal components of microparticles include platelet-derived mature mRNAs, pre-mRNAs, microRNAs (miRNA), and long non-coding RNAs (lncRNA) that can influence tumor cell gene expression and the crosstalk between cells. Additionally, microparticles containing cytoplasmic proteins and chemokine receptors contribute to the reinforcement of tumor cell adhesions and augmentation of the migratory behaviour of the recipient cells [51].

Microvesicle-derived RNAs have emerged as major contributors to the platelet-tumor cell crosstalk and the crosstalk with non-malignant cells in the TME, suggesting that these RNAs are important factors in cancer development.

Beyond transcriptional regulation, miRNAs exert significant influence on cancer progression by post-transcriptional mechanisms. Notably, miRNA binding to target mRNAs in the cytoplasm can trigger mRNA degradation or temporary inhibition of translation [52].

For example, a prominent role for miRNA-320a in pancreatic malignancy. Its overexpression is associated with a pro-tumorigenic phenotype, characterized by enhanced EMT, p53 downregulation, and increased proliferation, invasion, metastasis, and therapeutic resistance. In pancreatic cancer, the overexpression of miRNA-320a strongly contributes to EMT, downregulating of apoptosis [53], proliferation, invasion, metastasis, and drug resistance.

Long non-coding RNAs (lncRNAs) exert a diverse repertoire of regulatory functions encompassing epigenetic modifications, transcriptional and post-transcriptional control, and microRNA (miRNA) regulation. Additionally, lncRNAs participate in various cancer-associated signaling pathways, including those involving p53, AKT, and Notch, and influence a multitude of biological processes such as tumor proliferation, metabolism, and apoptosis [53]) [54].

Engaging with receptors on the tumor cell surface, platelets start rolling over the tumor cell, generating an augmented number of contact points that culminate in firm adhesions. The selectins, recognized as pivotal adhesion factors, play a crucial role in mediating the initial interactions between platelets and tumor cells.

The expression of selectins, especially the P-selectins on platelets and E-selectins on endothelial cells, may be induced by tumor cell-derived cytokines, contributing to the rolling motion of tumor cells on activated endothelium in the target area, a process that ultimately leads to extravasation [55].

Integrins, another class of adhesion molecules, play a pivotal role in establishing robust adhesions, particularly between αIIbβ3 (also recognized as GPIIb/IIIa) on platelets and αvβ3 and α5β1 on tumor cells. As highly versatile proteins, integrins act as essential connectors, facilitating interactions between cells and extracellular matrix ligands such as collagens, fibronectin, and laminin. The ability to integrate and transmit signals bidirectionally across plasma membranes underscores the multifunctional significance of integrins in orchestrating cellular interactions within the complex microenvironment.

#### 1.4.1 **Thrombospondin-1** (TSP-1)

A multi-domain extracellular matrix protein secreted upon platelet activation [56], binds to CD36 and CD47 receptors on tumor cells. This interaction plays an important role in mediating the formation of CTCs clusters [57] as well as the metabolic reprogramming of tumor cells [50,58,59].

TSP-1 also promote tumor cell migration by binding to cell surface receptors and ligands in the ECM [60]. TSP-1 induces the secretion of cytokines and growth factors such as TGF-β, VEGF, and PDGF, further enhancing tumor progression. Moreover, TSP-1 suppresses cytotoxic immune responses mediated by cytotoxic T cells and natural killer (NK) cells.

Platelet-derived growth factors (PDGFs), including TGF-β and VEGF, stimulate EMT, neo-angiogenesis, and ECM degradation via serine proteases and matrix metalloproteinases [[61], [62], [63]].

As migrating tumor cells progress through the endothelium, they employ cytoplasmic protrusions known as invadopodia. These structures are enriched with ECM-degrading enzymes, facilitating their infiltration between endothelial cells. This maneuver allows the tumor cells to breach endothelial junctions, transmigrate paracellularly, and gain access to the circulation [[64], [65], [66]]. Adenine nucleotides released from TAPs induce openings in the endothelial barrier, facilitating the migration of tumor cells through the endothelium. This effect is suggested to be primarily mediated by the activation of the endothelial P2Y2 receptor through ATP [12].

In summary, TAPs orchestrate a complex cascade of events that facilitate metastasis. TAPs contribute to tumor cell priming, adhesion, migration, and invasion, while also promoting the formation of a favorable metastatic niche.

### 1.5 Clustering and platelet protection of CTCs

Despite the presence of thousands of circulating tumor cells in many patients with cancer, only a small fraction of these cells survive and progress to form metastases. The immune system destroys most single CTCs and the adherence of platelets to CTCs constitutes an important protection against immune destruction. Platelets are first responders to intravasating tumor cells and can form a protective shield around them within seconds [67].

Some CTC/platelet aggregates form clusters with immune cells and stromal cells from the TME, e.g. pro-tumorigenic neutrophils, myeloid-derived suppressor cells (MDSC), cancer-associated fibroblasts and tumor-associated macrophages. It is estimated that the probability of metastasis formation from these clusters is 50 times higher compared to single CTCs [68,69] and further, if covered by CLEC-2 positive platelets, the proliferation, survival, and immune evasive capabilities are significantly increased [70].

In vitro and in vivo experiments have suggested several mechanisms for the adhesion between platelets and CTCs among others by CD97, an adapter protein that stimulates bidirectional signaling through the bridging of platelets and CTCs [71]. Furthermore, ADP binding to purinergic platelet receptors plays a crucial role in the granule secretion of bioactive molecules such as serotonin, TXA2, and PDGF contributing to platelet aggregation and cluster formation.

In the microenvironment of the blood, CTCs are prone to anoikis, a special form of programmed cell death occurring in anchorage-dependent cells when detached from their ECM. Furthermore, if CTCs lack “self” surface proteins, such as the “don't eat me” integrin-associated protein CD47 and the major histocompatibility complex-1 (MHC-1), CTCs are regarded as “non-self” and only survive shortly in the circulation [72,73]. However, important survival mechanisms are facilitated through platelet αvβ3 surface integrins that cross-link platelets and CTCs and mediate resistance to anoikis [74,75].

In a clinical study on metastatic prostate cancer, direct imaging liquid biopsy high-definition single cell assay showed that 97.5 % of patients had CTCs of which 30.4 % were positive for platelet-coating. These patients had by far the shortest overall survival of 8.2 months [72,76].

Upon platelet coating, CTCs may form pseudopodia around the platelets and create membrane fusions promoting the transfer of platelet-specific molecules onto the tumor cell surface [77,78]. Relevant examples of the survival strategies of CTCs are the transfer of MHC-1 and CD47 molecules [79,80]. Whereas the appearance of MHC-1 complexes on the CTCs surface inhibits NK cell activation, CD47 prevents phagocytosis by binding to the signal-regulatory protein alpha (SIRPα) on macrophages [81].

The MHC-1 presents fragments of intracellular peptides allowing CD8 T cells to identify and eliminate cells expressing mutated proteins. As a survival mechanism, tumor cells may down-regulate or shed MHC-1 and become less stimulatory or invisible to CD8 T cells. This, however, makes them more susceptible to NK cell-mediated killing. This balance is an essential part of the body's immune surveillance.

Intriguingly, CD47 expression on circulating tumor cells (CTCs) has been linked to breast cancer recurrence and metastasis [57,66]. Furthermore, by releasing TGF-β and PDGF, platelets contribute to immune evasion through downregulation of NKG2D surface receptors (immune checkpoint proteins) on natural killer (NK) cells, thereby diminishing their cytotoxic activity [82,83].

Intratumoral and circulating TAPs, akin to their associated stromal cells such as cancer-associated fibroblasts and tumor-associated macrophages, can express programmed cell death ligand 1 (PD-L1). This PD-L1 expression by TAPs contributes to the negative regulation of immune cell function within the tumor microenvironment. This fact explains why the effect of checkpoint inhibitor therapy using anti-PD-L1 does not solely depend on tumor cell PD-L1 expression. Incubation of PD-L1 positive platelets with PD-L1 negative tumor cells resulted in the appearance of PD-L1 on tumor cell surfaces, a reaction that was significantly reduced by the anti-platelet effects of aspirin exposure [84].

### 1.6 CTCs arrest, endothelial adhesion and extravasation

The metastatic capability of CTCs is regulated by their capacity to overcome the forces within intravascular flow [85] and to adhere to the endothelium. This process is governed by a myriad of adhesion molecules present on both CTCs clusters and endothelial cells.

**Fig. 3:**
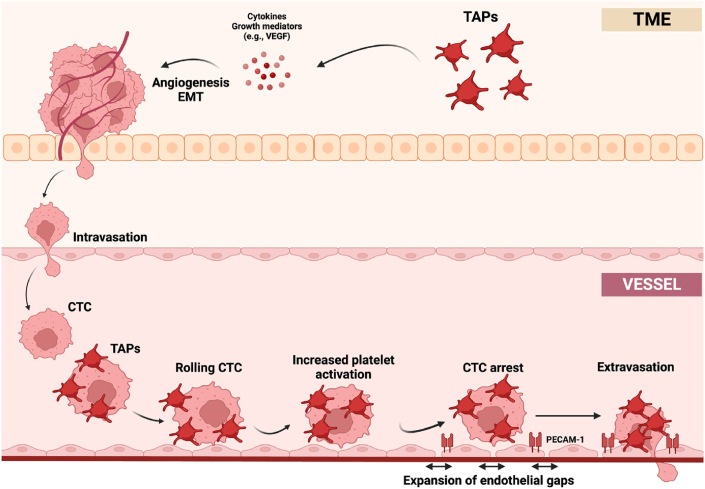
In the TME, TAPs secrete cytokines and growth mediators stimulating hemostasis, angiogenesis and cell adhesion. These signals promote EMT and the intravasation of cancer cells. Once in the bloodstream, CTCs can adhere to the vessel wall and extravasate to a secondary site with the assistance of platelets. Rolling along the vessel wall, platelets attached to CTCs further activate and increase the expression of PECAM-1. PECAM-1 and integrins help slow down the CTCs while expanding the endothelial gaps and easing the migration and invasion of CTCs. CEP: Cancer-educated platelets, CTCs: circulating tumor cell, EMT: epithelial-mesenchymal transition. PECAM-1: platelet endothelial cell adhesion molecule 1. TAPs: tumor-activated platelet. TME: tumor microenvironment. Figure made on biorender.com.

The margination of CTCs in small vessels (Fig. 1) is considered a prerequisite for CTCs adhesion and is similar to the margination of immune cells that travel close to the vessel wall. As the concentration of red blood cells is highest around the centerline of the vessel, CTCs and white blood cells are forced towards the vessel wall. In addition to this wall-directed deviation, the contact with the vessel wall depends on the stiffness of CTCs and the presence of receptors and ligands on clusters and endothelial cells [86]. Approaching the vessel wall, the cluster initiates a rolling motion along the wall (Fig. 3), generating a vortex of plasma and shear force in the interplay between the cluster and the vessel wall. Interestingly, the vortex further attracts and activates more platelets to the CTCs [87], which decreases the rolling motion and augments stable adhesion to the endothelium [87]. Activation of the CTCs-attached platelets increases the expression of platelet endothelial cell adhesion molecule-1 (PECAM-1), a ligand to integrin αVβ3. PECAM-1 and integrins slow down the speed of the CTCs and also contribute to the expansion of the passageway between endothelial cells (Fig. 3) [85,88].

Further to the direct effects on the proliferation and invasion of CTCs, PECAM-1 is an immune checkpoint molecule that negatively regulates the cytotoxicity of monocytes and macrophages [71,89].

The C-type lectin-like receptor 2 (CLEC-2) on the surface of platelets interacts with podoplanin on the tumor cells and facilitates EMT and extravasation [90]. Intravenous injection of podoplanin-positive lung tumor cells into mice lacking CLEC-2 or treated with anti-podoplanin antibodies failed to establish metastasis [91].

Finally, in vitro and in vivo assays revealed that human and mouse platelet α6β1 supported platelet adhesion to various types of tumor cells. Directly binding to platelet α6β1, a disintegrin and a metalloprotease 9 (ADAM9) was identified as the major receptor of α6β1 and this interaction efficiently promoted platelet activation, tumor cell extravasation, and lung metastasis [85].

Many tumor cell types exhibit overexpression of ADAM9, a transmembrane metalloproteinase. This enzyme possesses a dual functionality: the extracellular disintegrin domain mediates cell adhesion, while its metalloprotease domain facilitates the shedding of various cell surface proteins through ectodomain cleavage.

To enhance anticancer treatment, agents such as sorafenib and regorafenib are used to reduce the expression of ADAM9, inhibit tumor cell shedding of MHC-1, and activate NK cells. Directly targeting α6β1 efficiently impaired tumor metastasis and may represent a promising therapeutic target [92].

Platelets promote tumor cell extravasation immediately after vascular arrest, a critical period in which the endothelial barrier has to be opened and penetrated. As mentioned, platelets interact with CD97 on the surface of the CTCs leading to granule release. Platelet ADP secretion, activation of endothelial purinergic receptors and subsequent activation of protein kinase C induce the opening of the endothelial barrier [12,93] which in turn facilitates the extravasation of CTCs. The additional activation of the purinergic receptors on CTCs has an oncogenic by accelerating the cell cycle and mediating the expression of EMT/invasion-related genes including IL-8, E-cadherin, Snail and Claudin-1 [94]. The purinergic receptors have been found overexpressed in colon cancer [95], pancreatic cancer [96] and prostate cancer [97]. Following transmigration, the CTCs reach the premetastatic niche and a micrometastasis has been established (Fig. 3)

### 1.7 Platelets in the pre-metastatic niche

Concurrently with their supporting role in the dissemination of CTCs, TAPs play crucial roles in the formation of the pre-metastatic niche, the permissive microenvironment that nurtures the arriving CTCs and secures their growth. TAPs release growth factors including VEGF and TGF-β and inflammatory cytokines and chemokines such as CXCL5 and CXCL7 to recruit pro-tumorigenic immune cells including MDSC to drive the immune evasion (Fig. 1) [98]. TAPs may even take up growth factors and enzymes, e.g. metalloproteinases, from the primary cancer and release them in the pre-metastatic niche [99].

The expansion of the MDSC population in the bone marrow is a result of tumor-released growth factors and inflammatory mediators derailing myelopoiesis. As solid adhesions with arriving tumor cells are established, platelets secrete growth factors, PDGF, VEGF, and TGF-β to recruit MDSC to support survival and invasion [100,101]. The MDSC promote tumor cell proliferation by delivering energy-rich lipid vesicles and by suppressing the cytotoxic anticancer immune responses [102,103].

Platelet P-selectin (CD62P), a cell adhesion molecule crucial for cellular interactions, translocates to the platelet surface upon activation. This translocation enables engagement with specific carbohydrate receptors (P-selectin glycoprotein-1 or PSGL-1) (Fig. 2) on neutrophils, monocytes, certain tumor cells, and tumor-associated macrophages, thereby fostering their recruitment to the premetastatic niches and supporting the establishment of a protumorigenic environment [104]. Further, P-selectin contributes to the platelet coating of CTCs, the systemic dissemination of tumor cells in vivo [105] and metastasis formation in vivo [106] and mediates platelet infiltration into tumors [107].

### 1.8 Clinical aspects and potential treatment targets

Platelets play a significant role in the progression of cancer, and various diagnostic and therapeutic strategies are consequently based on this aspect of tumor biology.

#### 1.8.1 Platelet counts

The cancer-associated thrombocytosis and platelet/lymphocyte ratio often correlate with chemoresistance and disease progression and predict poor survival outcomes [108,109].

Thrombocytosis has been observed in some patients prior to diagnosis, particularly those with colon, lung, ovarian, and gastric cancers [110].suggesting that platelet counts could potentially serve as an early marker of these cancers. However, normal platelet levels may not preclude tumor growth, and low platelet counts (thrombocytopenia) have also been linked to worse outcomes [110,111,112]. These findings indicate that both the quantity and quality of platelets could influence cancer prognosis.

Furthermore, the levels of platelet/leukocyte aggregates (PLAs) have been correlated to high P-selectin expression on platelets [113]. In patients with lung cancer, higher quantities of PLAs have been observed when compared to healthy volunteers [114]. Measurement of PLAs could be a relevant biomarker of disease progression and aggressiveness in cancer.

#### 1.8.2 RNA-profiles

Interaction with tumor cells results in altered platelet mRNA profiles. A recent study demonstrated that platelets exposed to a conditioned medium from CTC lines exhibited accelerated activation compared to controls. This activation was accompanied by significant transcriptomic alterations associated with metastasis, including genes involved in inflammation, hypoxia, EMT, apoptosis, and signaling pathways like TNFα, PI3K/AKT/mTOR, and mTORC1 [98]. The uptake of mutant or tissue-specific RNA transcripts and abnormally spliced RNA by platelets enables a clear distinction between RNA profiles of normal platelets and TAPs which may improve outcome predictions [4].

In a prospective trial where blood was collected from 136 patients with cancer and 39 healthy donors, mRNA sequencing of TAPs differentiated between six primary tumor types with 71 % accuracy and distinguished cancer patients from healthy individuals with 96 % accuracy [38]. Building on recent findings, the modulation of the platelet transcriptome in cancer patients holds promise as a potential diagnostic tooll [115]. Interestingly, the normalization of the platelet proteome following surgical tumor resection underscores the potential of platelet proteomics for not only diagnosing cancer but also monitoring the effectiveness of treatment regimens and detecting disease recurrence [116].

#### 1.8.3 Surgical interventions

During surgical intervention, platelets are activated by the surgical stress response through the platelet TLR4-dependent ERK5/GPIIb-IIIa pathway that leads to increased platelet/CTCs clusters [117] and impacts the number of CTCs. Given the established role of platelets in shielding CTCs from immune attack and mechanical stress, persistent thrombocytosis following surgery might promote CTC dissemination and worsen clinical outcomes [[118], [119], [120]].

A systematic review and meta-analysis encompassing 18 prospective studies and 1321 patients with NSCLC demonstrated a positive association between elevated postoperative CTC clusters and risk of both disease recurrence and mortality [121]. CTCs were detected in the pulmonary vein in a high number (90 %) of patients during surgery and less in the peripheral veins (40–80 %). CTCs clusters could be demonstrated in 50 % of patients [122,123].

In a murine colon cancer model, the formation of CTCs clusters increased the entrapment by neutrophil extracellular traps and promoted the formation of distant metastasis [113].

#### 1.8.4 Potential future compounds

Finally, we want to draw attention to two compounds, PAR-1 inhibitors and phosphodiesterase inhibitors (PDI) that may be part of the anticancer drugs of the future. Elevated thrombin, which is linked to worse cancer outcomes, activates PAR-1 receptors on both platelets and cancer cells [124] which promote tumor growth, invasion, and survival [125] as shown with the progression of KRAS mutant CRC via the PAR1-PDK1-AKT signaling pathway [126].

Despite potential side effects, PAR-1 inhibitors show promise in reducing both platelet aggregation and metastasis formation, ongoing research explores their use as adjuvant cancer therapy [124,127,128].

The phosphodiesterase enzymes (PDE) degrade cyclic AMP and promote tumor growth by increasing mTORC1 signaling [129].

Targeting PDE with phosphodiesterase inhibitors (PDI) elevates cyclic AMP levels, effectively blocking mTORC1 and hindering tumor development. Besides, PDI therapy has emerged as a successful therapeutic approach for various inflammatory conditions including chronic obstructive pulmonary disease, astma, and psoriasis. PDI are not yet approved in cancer therapy, but ongoing research is exploring their effects on cancer cell signaling[130]^.^

#### 1.8.5 Antiplatelet agents

Antiplatelet therapies are proposed as a strategy to disrupt signaling events that drive disease progression and to prevent TE. Drugs targeting platelet aggregation such as COX-inhibitors (e.g. Aspirin) and P2Y12-inhibitors (e.g. Clopidogrel) have been studied for their potential anticancer effects. COX inhibitors block the production of mitogenic and anti-apoptotic prostaglandins, whereas P2Y12 inhibitors block the binding of ADP to the P2Y12 receptor and prevent platelet aggregation. In a meta-analysis, patients who took aspirin after surgery for CRC were less likely to develop metastases [131]. The primary established effect of aspirin is impaired platelet aggregation via inhibition of platelet thromboxane A_2_ synthesis being caused by an unselective, irreversible COX inhibition that blocks the arachidonic acid metabolism and prostaglandin production. Inhibition of both isoenzymes (COX-1 and COX-2) is considered important for the antitumorigenic effect. In a murine model, it was demonstrated that COX-1 is highly expressed in platelets infiltrating the mucosa and that the platelet COX-1 pathway is critical to the development of intestinal neoplasia through upregulation of COX-2 [132].

Other mechanisms of aspirin are the down-regulation of the NF-kappa B signaling pathway, inhibition of toll-like receptors [133], and abrogation of the release of platelet VEGF [6].

In patients with CRC, PIK3CA mutations and high COX-2 expression emerged as potential positive prognostic factors for survival [134] However, further investigation through randomized controlled trials is crucial to evaluate the efficacy and safety of adjuvant and neoadjuvant aspirin therapy in CRC management. ^134134^At present, clinicaltrials.gov registers only a limited number of prospective, randomized, phase 3 trials investigating platelet function and its impact on solid cancers, predominantly focusing on Aspirin (NCT02804815, NCT03464305, NCT02647099).

The P2Y12 receptor is a G protein-coupled receptor that is activated by ADP. By blocking the P2Y12 receptor the risk of thrombosis is reduced. In addition, the P2Y12 on tumor cells contribute to EGFR activation and the expression of SLUG and ZEB1, two transcriptional factors implicated in chemoresistance and metastasis. In a pancreatic ductal adenocarcinoma cell line study, P2Y12 inhibition with ticagrelor significantly reduced EGF-dependent AKT activation and increased the anticancer activity of anti-EGFR treatment. Further, ticagrelor significantly decreased the proliferation of cancer cells but not normal pancreatic cells. In a rodent model, the combination of ticagrelor and gemcitabine significantly reduced tumor growth. Also, synergism was observed when ticagrelor was combined with various chemotherapeutics. The drugs alone had a minimal effectP2Y12 inhibitors include ticagrelor (Brilique) and prasugrel (Effient) are already approved for prevention of cardiovascular events, and clinical trials are investigating the impact on survival in cancer. In a preclinical study of pancreatic cancer demonstrated that the combination of ticagrelor with gemcitabine exhibited synergistic effects which led to significantly reduced tumor growth compared to minimal effects observed with either gemcitabine or ticagrelor as monotherapies^135.^ Nonetheless, P2Y12 therapy (e.g. Clopidogrel), like other anti-aggregation treatments, is associated with an increased risk of bleeding. In a meta-analysis of randomized trials including 24,325 participants, P2Y_12_ inhibitor therapy was compared to aspirin for the prevention of cardiovascular events. The primary outcome was a composite of cardiovascular death, myocardial infarction, and stroke. Secondary outcomes were major bleeding and net adverse clinical events. The risk of the primary outcome and net adverse clinical events were lower with P2Y_12_ inhibitors compared with aspirin over 2 years (HR: 0.88; 95 % CI: 0.79–0.97; P = 0.012) and HR: 0.89; 95 % CI: 0.81–0.98; P = 0.020, respectively), but the risk of major bleeding was similar (HR: 0.87; 95 % CI: 0.70–1.09; P = 0.23) [136].

#### 1.8.6 Antiplatelet antibodies

Glycoprotein IIb/IIIa receptor antibodies, exemplified by the Fab fragment derived from monoclonal antibody 7E3 (Abciximab), are primarily employed to avert cardiac events during percutaneous coronary intervention. They are also utilized for preventing myocardial infarction in patients with unstable angina unresponsive to conventional treatments and for thrombolysis. The ligands of platelet GPIb and GP IIb/IIIa integrin receptors include vWF, thrombin, P-selectin, fibrinogen, and thrombospondin. These molecules play crucial roles in platelet aggregation and the metastasis-promoting activity of platelets, rendering GPIb and GP IIb/IIIa attractive targets. Monoclonal antibodies targeting both receptors have been investigated as a means to disrupt and prevent the formation of platelet-tumor cell aggregates and metastasis, demonstrating effectiveness in preclinical models [137,138]. However, while antiplatelet antibodies may hold promise as therapeutic agents, their use is currently limited due to the associated risk of bleeding complications. Consequently, they are not yet part of active clinical trials [139].

#### 1.8.7 Inhibition of platelet**-**activating factors

Platelet activation and subsequent platelet-tumor cell adhesion is triggered through a diverse array of factors released by tumor cells, including thrombin, ADP, and vWF. Targeting these platelet-activating factors or their receptors may disrupt platelet-tumor cell adhesion and inhibit cancer progression. In an animal model on brain cancer and by using multiphoton laser-scanning microscopy, it was demonstrated that vWF-mediated platelet accumulation promoted the metastatic cascade and further, that anticoagulation with light-weight heparin or Pradaxa reduced intravascular tumor cell arrest and reduced metastasis formation [140].

Therapeutically targeting platelet-tumor cell adhesion holds promise as a synergistic strategy when combined with established anticancer modalities, including chemotherapy, immunotherapy, or anti-angiogenic agents. This integrated approach has the potential to significantly enhance treatment efficacy by suppressing tumor growth and metastasis, ultimately improving patient outcomes. While promising, it is important to note that the development of targeted therapies against platelet-tumor cell adhesion is still in the early stages, and more research is needed to optimize these strategies. Furthermore, the efficacy and safety of these approaches may vary depending on the specific tumor type, patient population, and other factors. Clinical trials are underway to further investigate the potential of targeting platelet/tumor cell adhesion as a therapeutic strategy in cancer.

#### 1.8.8 Platelets as drug carriers

Platelets hold promise as a drug delivery platform for cancer treatment. Tumors often exhibit abundant vascular endothelial growth factor (VEGF), promoting abnormal and leaky vasculature (heteromorph vascularization). The leaky endothelium facilitates direct interaction between platelets and tumor cells within the tumor microenvironment (TME). A particularly promising application lies in leveraging platelets as targeted carriers of chemotherapeutic agents. Their limited expression of ABO antigens and complete lack of Rhesus antigens further minimize potential adverse immune reactions.

For example, Doxorubicin-loaded platelets significantly inhibited cancer growth in vivo and in vitro [143,144]. Antibodies such as anti-pd-1 may be conjugated onto the platelet membrane to inhibit the PD-1 and PD-L1 driven T cell exhaustion and immune evasion. In a mouse model of prostate cancer metastasis, genetically engineered platelets expressing the tumor necrosis factor-related apoptosis-inducing ligand (TRAIL) were demonstrated to kill cancer cells and significantly reduce metastases [144]. In a murine leukemia model platelets decorated with anti-PD-1 antibodies (aPD-1) and conjugated to hematopoietic stem cells suppressed the growth of leukemia cells and enhanced the anti-leukemia immune responses by an increase in the number of T cells. The production of cytokines and chemokines significantly increased and prolonged the survival time of the mice [141].

Although multiple animal models have demonstrated therapeutic efficacy due to improved pharmacokinetics, cancer cell targeting, drug uptake, and slow drug elimination, no platelet-based drug delivery systems have entered clinical trials. More research is needed to determine the clinical relevance of this technology. Some of the major obstacles are the short lifespan, drug loading and release, immunogenicity, and standardization of production methods [142,145].

Table 1 summarizes the preclinical and clinical trials examining the potential of targeting platelets in cancer treatment that have been discussed in this section.

**Table 1:** Overview of the preclinical and clinical trials mentioned in the text.

Author	Year	Study type	Cancer type	Intervention	Findings
**Preclinical studies**					
**Bruno et al.** [132]	2022	Mouse models	Intestinal cancer	Deletion of platelet COX-1	Deletion of platelet COX-1 reduced the number and size of tumors in the small intestine.
**Feinauer et al.** [140]	2021	Cell lines Mouse models	Breast cancer Malignant melanoma	Low molecular weight heparine or dabigatran + anti vWF antibody.	The intervention prevented clot formation, cancer cell arrest, extravasation, and the formation of brain macrometastases.
**Elaskalani et al.** [135]	2020	Cell lines	Pancreatic cancer vs. normal pancreatic cell lines	Ticagrelor (P2Y12 inhibitor) with chemotherapeutics	Ticagrelor decreased the proliferative capacity of pancreatic cancer cells.
**Qi et al.** [137]	2018	Mouse models	Myeloma cell lines	1D12 and 2B4 binding to GPIbα aa 41–50 and aa 277–290 respectively thereby affecting von Willebrand factor (vWF)-GPIbα interaction.	The binding inhibited the interaction among platelets, tumor cells, and endothelial cells (in vitro) and reduced the mean number of nodules in the experimental and spontaneous metastasis models (in vivo).
**Hu et al.** [141]	2018	Mouse models	Leukemia	Hematopoietic stem cells (HSCs) conjugated to anti-PD-1 antibody-decorated platelets (aPD-1 platelets)	aPD-1 platelets suppress the growth and recurrence of leukemia in mice.
**Xu et al.** [142]	2017	Mouse models	Lymphoma	Doxorubicin was loaded in natural platelets.	Doxorubicin loaded platelets improved the anti-tumor activity of doxorubicin by regulating the expression of apoptosis-related genes.
**Sarkar et al.** [143]	2013	Cell lines and mouse models	Lung cancer	Platelets as delivery agents for doxorubicin hydrochloride therapy.	The cytotoxic effects of doxorubicin hydrochloride were significantly higher platelets loaded with the drug compared to the free drug.
**Clinical studies**					
**Mädge et al.** [134]	2022	Meta analysis	Colorectal cancer	Aspirin	Aspirin use after diagnosis was associated with an improvement in CRC-specific survival with a hazard ratio (HR) for cancer-related death of 0.74 (95 % CI: 0.62–0.89).
**Algra et al.** [133]	2012	Systematic review	Gastrointestinal cancers	Aspirin	Regular use of aspirin reduces the long-term risk of several cancers and the risk of distant metastasis.
**Rothwell et al.** [131]	2012	Pooled RCTs	Solid cancers	Aspirin ≥75 mg daily	Reduced risk of cancer with distant metastasis.

## 2 Discussion and future perspectives

Platelets are not mere blood clotting components but rather active participants in the metastatic cascade. To increase proliferation and survival, human tumor cells promptly bind to platelets and activate them through surface receptors. Through the release of various growth factors, activated platelets promote EMT and all stages of disease progression including immunosuppression in the TME and systemically.

Despite challenges [146], targeted interventions against platelets hold immense promise for cancer treatment. By disrupting platelet-tumor cell interactions and modulating platelet-derived factors, we may potentially halt metastasis and improve patient outcomes. Notably, combinations of antiplatelet agents with conventional chemo- or immunotherapy have shown to dramatically improve therapeutic efficacy in preclinical experiments – a path that should be explored in clinical trials. Also, future research should focus on developing novel and selective platelet-targeted therapies that minimize the risk of bleeding complications. By selectively disrupting the adhesive interactions facilitated by P-selectin, it may be possible to impair the pro-metastatic effects of platelet-tumor cell interactions and impede the dissemination of tumor cells. Targeting thrombospondin may represent a potential strategy to inhibit platelet-cancer cell interactions and suppress disease progression. Loading nanoparticles onto the surface, platelets may be used as drug carriers and for imaging purposes and drug-loaded platelet exosomes may enhance tumor-specific drug accumulation [147].

Antiaggregating drugs like aspirin, the only non-selective and irreversible COX inhibitor, and the selective and reversible COX2 inhibitors such as celecoxib and rofecoxib have been investigated in clinical trials in the context of prevention and reduction of recurrence as they may positively impact survival measures [148], however, conflicting outcomes are reviewed in Refs. [149,150]. The main concerns of COX-inhibition are side effects in terms of bleeding and increased risk of cardiovascular events, especially of the selective COX-2 inhibitors. Thus, the focus has turned to disrupt tumor cell-platelet interactions by inhibition of the platelet ADP receptor P2Y12 that leads to reduced aggregation and may impact the metastatic cascade. Anti-podoplanin and anti-CLEC-2 drugs may be effective therapies for inhibiting tumor growth and dissemination [151,152]. However, clinical evidence is lacking.

In addition, anti-platelet therapy could be an important prophylaxis against TE in patients diagnosed with advanced cancer. With progressing disease, however, patients may develop thrombocytopenia due to chemotherapy, immune destruction and bone marrow infiltration, impairing normal hemostasis and increasing the risk of bleeding.

Finally, platelets could serve as diagnostic and prognostic biomarkers for cancer to support diagnosis and monitor cancer. Clinical research has demonstrated that platelet RNA profiles exhibit high diagnostic accuracy, surpassing basic platelet characterization, and hold potential for outcome predictions. These findings necessitate prospective validations in larger cohorts before establishing clinical utilities.

## Ethics approval and consent to participate

N/A. The manuscript is a review paper, the point is not relevant.

## Consent for publication

N/A. The manuscript is a review paper, the point is not relevant.

## Data availability statement

N/A. This is a review paper; only bibliographical data were used (see reference list). No data in this study have been deposited into a publicly available repository.

## Funding information

No funding was received.

## CRediT authorship contribution statement

**Hans Raskov:** Writing – review & editing, Writing – original draft, Visualization, Validation, Supervision, Investigation, Conceptualization. **Adile Orhan:** Writing – review & editing, Writing – original draft, Visualization, Validation, Conceptualization. **Mette Ørskov Agerbæk:** Writing – review & editing, Validation. **Ismail Gögenur:** Writing – review & editing, Validation, Supervision.

## Declaration of competing interest

The authors declare that they have no known competing financial interests or personal relationships that could have appeared to influence the work reported in this paper.
